# Recent advances in ocular graft-versus-host disease

**DOI:** 10.3389/fimmu.2023.1092108

**Published:** 2023-01-25

**Authors:** Xianjing Cheng, Ruihao Huang, Shiqin Huang, Wei Fan, Rongdi Yuan, Xiaoqi Wang, Xi Zhang

**Affiliations:** ^1^ Medical Center of Hematology, Xinqiao Hospital, State Key Laboratory of Trauma, Burns and Combined Injury, Army Medical University, Chongqing, China; ^2^ School of Medicine, Chongqing University, Chongqing, China; ^3^ Department of Ophthalmology, Xinqiao Hospital, Army Medical University, Chongqing, China; ^4^ Jinfeng Laboratory, Chongqing, China

**Keywords:** ocular graft-versus-host-disease, clinical characteristics, the pathogenic mechanism, novel therapeutic targets, tear biomarkers

## Abstract

Ocular graft-versus-host-disease (GVHD) remains a significant clinical complication after allogeneic hematopoietic stem cell transplantation. Impaired visual function, pain, and other symptoms severely affect affected individuals’ quality of life. However, the diagnosis of and therapy for ocular GVHD involve a multidisciplinary approach and remain challenging for both hematologists and ophthalmologists, as there are no unified international criteria. Through an exploration of the complex pathogenesis of ocular GVHD, this review comprehensively summarizes the pathogenic mechanism, related tear biomarkers, and clinical characteristics of this disease. Novel therapies based on the mechanisms are also discussed to provide insights into the ocular GVHD treatment.

## 1 Introduction

Allogeneic hematopoietic stem cell transplantation (allo-HSCT) represents the only curative treatment for some hematologic non-malignant and malignant diseases. With the improvements in HSCT technology, a growing number of patients with hematological diseases have longer life expectancies after allo-HSCT (1, 2). However, chronic graft-versus-host disease (cGVHD) is one of the most common complications after HSCT, occurring in 30% to 70% of patients who undergo HSCT and impairing their quality of life (3). cGVHD is a syndrome with variable clinical manifestations resembling autoimmunity, and it can involve several organ systems. For example, ocular GVHD affects 40–60% of patients who undergo allo-HSCT and occurs more frequently in patients with cGVHD of any organ system (60–90%) (4). Similar to cGVHD affecting other organ systems, ocular GVHD is predominantly characterized by T-cell-mediated inflammatory damage, which leads to ocular tissue damage and progressive fibrosis and exhibits various clinical features (5). The most common symptoms of ocular GVHD are dryness, ocular irritation, red eye syndrome, intermittent blurring of vision, photophobia, and ocular pain (6); other distinctive manifestations of ocular GVHD include gritty, cicatricial conjunctivitis; keratoconjunctivitis sicca; and confluent areas of punctate keratopathy (3). Although the changes in ocular GVHD have the potential to cause severe visual impairment and a substantial decrease in patients’ quality of life, the diagnosis of and therapies for ocular GVHD still represent a challenge due to the unclear pathogenic mechanisms resulting in ocular surface deterioration.

At present, there are two widely acknowledged international diagnostic criteria for ocular GVHD. The National Institutes of Health (NIH) revised their diagnostic criteria in consensus papers published in 2014. According to the NIH guidelines, ocular GVHD can be diagnosed by a low Schirmer’s test value, with a mean value of ≤ 5 mm at 5 minutes, or a new onset of keratoconjunctivitis sicca as determined by a slit lamp examination, with a mean Schirmer’s test value of 6 to 10 mm (3). In 2013, another International Consensus Group of ophthalmologists proposed a new set of diagnostic criteria for ocular GVHD based on subjective and objective clinical parameters, including the ocular surface disease index (OSDI), Schirmer’s score without anesthesia, corneal fluorescein staining, and conjunctival injection (7). However, Schirmer’s test without anesthesia lacks specificity and has poor reliability and sensitivity in diagnosing and monitoring ocular GVHD, particularly in milder cases (false-positive rate: 19.4% and false-negative rate: 36.4%) (8). Furthermore, the clinical diagnosis of ocular GVHD depends greatly on the observation of the symptoms and clinical signs and is thus without sufficient specificity. Moreover, the diagnosis is often made in the middle and late stages of ocular GVHD and is subjective. The inflammatory process in ocular GVHD can involve the entire ocular surface, and the current international diagnostic criteria do not include the effects of fibrosis in the eyelids, meibomian gland, and lacrimal duct system (9). Simultaneously, the criteria lack any indicators of immune-related and quantitative objective inflammatory factors to diagnose and assess the disease severity of ocular GVHD. Taken together, these issues show that the current international diagnostic criteria cannot yet fully address the clinical situation. In addition, the early recognition of and referral for ocular GVHD are challenging for hematologists.

**Figure 1:**
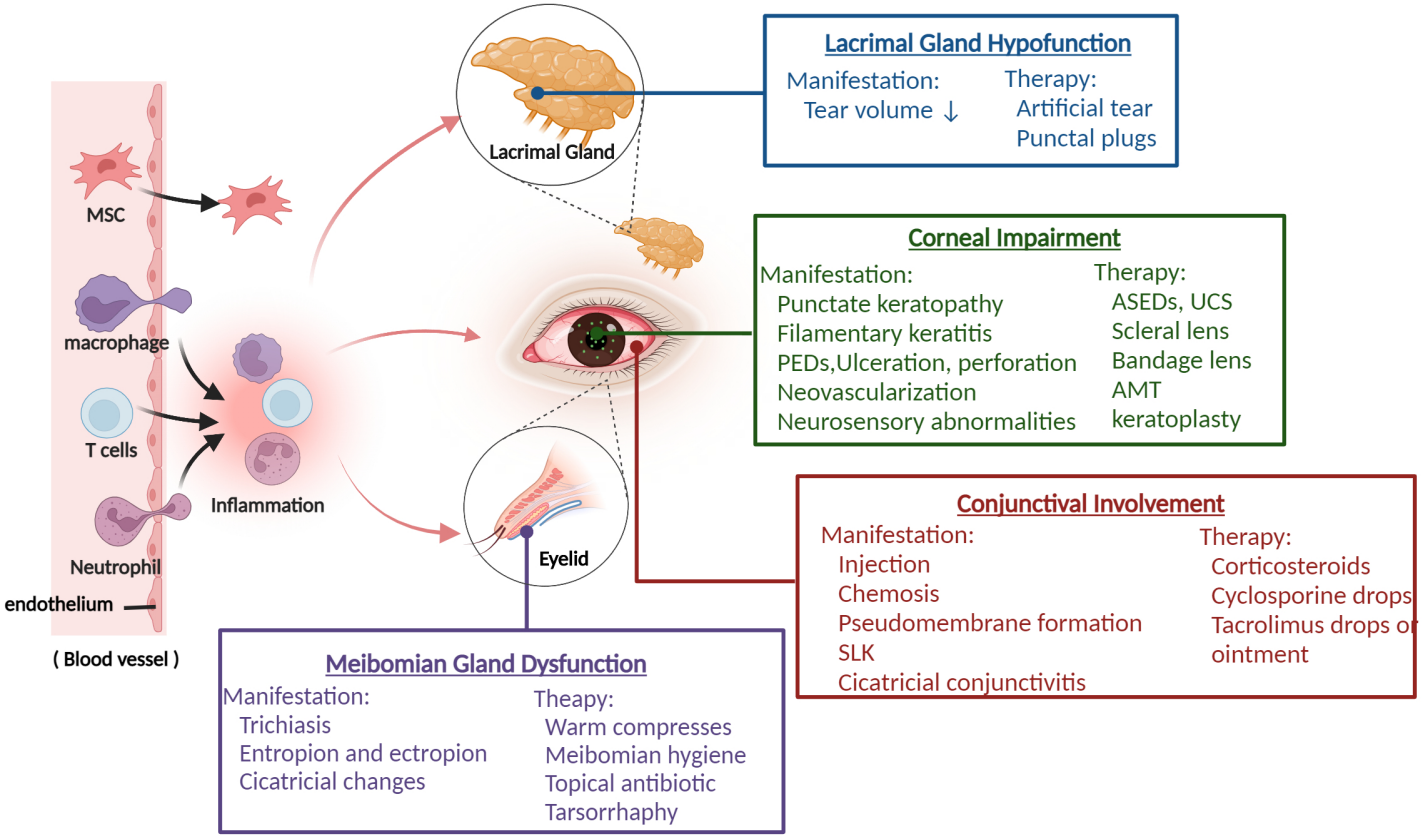
The overview of ocular GVHD and clinical management. Donor-derived immune cells (T cells, macrophage, neutrophil) cross the blood vessel barrier into the eye and drive the ocular inflammatory response, which leads to ocular tissue damage including lacrimal glands, meibomian glands, corneal, conjunctival and presents various related clinical characteristics. Based on the manifestations of different ocular impairments, it helps clinicians to take appropriate treatment measures. PEDs, persistent epithelial defects; ASEDs, autologous serum eye drops; UCS, umbilical cord serum; AMT, amniotic membrane graft; SLK, superior limbal keratoconjunctivitis. (Created with BioRender.com).

Due to the wide severity spectrum and the lack of randomized studies, there is currently no unified and approved topical treatment for ocular GVHD. The common therapies for ocular GVHD are mostly empirical and consist of lubrication, autologous serum eye drops, topical cyclosporine, tacrolimus, corticosteroids, and therapeutic lenses. Generally, comprehensive treatment, such as punctual occlusion, amniotic membrane graft, cornea grafts, and tarsorrhaphy, is required for patients with severe, persistent, or complex ocular GVHD, depending on the patient’s condition (10, 11) **(** **Figure 1** **)**. Unfortunately, many patients do not achieve adequate control despite these treatments. Thus, novel therapeutic strategies are urgently needed, particularly strategies based on the disease’s pathogenesis.

In this review, we summarize the pathogenic mechanism and related therapeutic new targets, as well as tear biomarkers, to provide insights into the clinical diagnosis and treatment of ocular GVHD.

## 2 Pathogenic mechanism and novel therapeutic targets

Typically, ocular GVHD involves the anterior segment of the eye, including the lacrimal gland (LG), conjunctiva, cornea, and meibomian gland. T-cell infiltration of these tissues initiates an immunoinflammatory cascade. Reportedly, the cascade response primarily consists of apoptosis induction, immune cell recruitment, and the production of inflammatory cytokines and chemokines (12). Thus, summarizing the underlying pathogenic mechanism in different parts of the ocular surface helps to understand the onset and development of ocular GVHD and facilitates the search for new therapeutic targets and treatment pathways.

### 2.1 Lacrimal gland hypofunction

**Figure 2:**
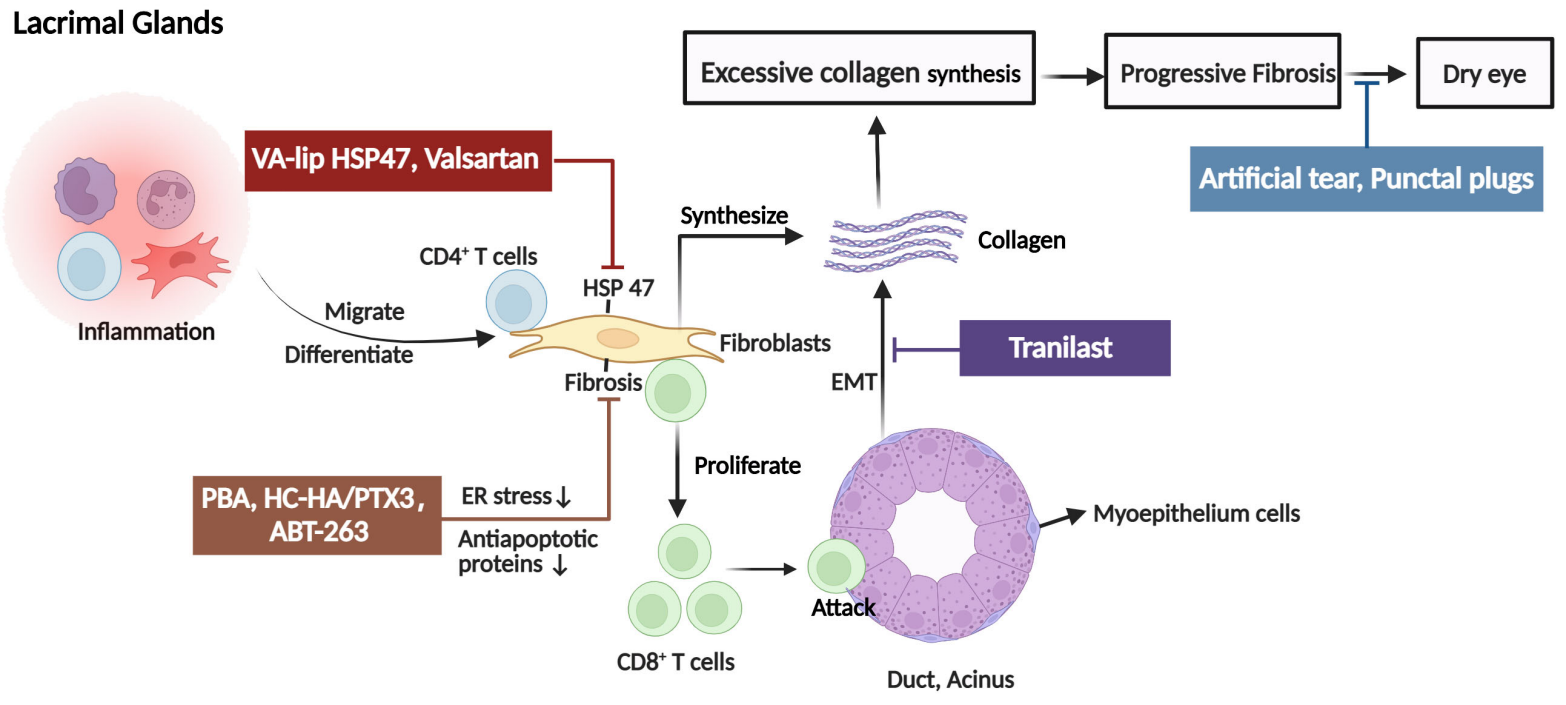
The pathogenic mechanism in the lacrimal glands (LG) of ocular GVHD and treatment. Donor CD4+ T cells and activated CD8+ T cells infiltrate the periductal area of the lacrimal gland causing tissue injury. In addition, fibroblasts with high expression of heat shock protein (HSP)47 are also activated and synthesize excessive collagen contributing to fibrosis in the LG and causing dry eye. The clinical therapies for ocular GVHD patients with dry eyes include artificial tears and punctal plugs. For novel therapeutic targets, it is reported that tranilast alleviates inflammation and retard fibrotic changes in LG pathology in cGVHD by preventing epithelial-mesenchymal transition. VA-lip HSP47 and Valsartan mainly reduce collagen synthesis by inhibiting or decreasing the expression of HSP47 fibroblasts. The mechanisms by which PAB, HC-HA/PTX3, and ABT-263 reduced fibrosis may involve fibroblasts. PAB specifically reduced endoplasmic reticulum stress induced by cGVHD in fibroblasts to alleviate fibrosis. HC-HA/PTX3 decreased the infiltration of fibroblasts to inhibit abnormal collagen synthesis. As for ABT-263, it selectively inhibited the antiapoptotic proteins (BCL-2 and BCL-xL), thereby mitigating the detrimental effects of senescent cells, including fibroblasts, on ocular GVHD. EMT, epithelial-mesenchymal transition; PBA, 4-phenyl butyric acid; HC-HA/PTX3, heavy chain-hyaluronan/pentraxin 3; ER, endoplasmic reticulum, VA-lip HSP4, vitamin A–coupled liposomes containing HSP47 small interfering RNA (siRNA) against HSP47. (Created with BioRender.com).

The LG is the primary target of ocular involvement in cGVHD. In ocular GVHD, donor CD4+ T cells and activated CD8+ T cells infiltrate the periductal area of the LG. Then, T cells caused tissue damage and generated a proinflammatory environment that is characterized by the recruitment of macrophages, Antigen-presenting cells (APCs), and additional T cells. In addition to the recruitment of immune cells, CD34+ stromal fibroblasts are also recruited and activated (13–15). The pathological fibroblasts are chimeric, and nearly half are of donor origin, probably derived from bone marrow mesenchymal stem cells (16, 17). Furthermore, these cells have low expression of α-smooth muscle actin (α-SMA) and high expression of heat shock protein (HSP)47, major histocompatibility complex class II, and costimulatory molecules (18–20). Therefore, fibroblasts with highly migratory and invasive properties could promote excessive collagen assembly in and around the periductal areas. Reportedly, fibroblasts also contribute to inflammation development by acting as APCs and interacting with lymphocytes in patients with ocular GVHD. Macrophages and fibroblasts also activate the endoplasmic reticulum stress pathway (21), and the tissue renin-angiotensin system (RAS) (22) synthesizes an excessive amount of extracellular matrix, resulting in rapid interstitial inflammation and fibrosis. In addition, study findings suggest a potential association between ocular GVHD pathogenesis in the LG and senescent cells through the senescence-associated secretory phenotype (SASP) and oxidative stress (23). Senescent cells produce cytokines and chemokines, such as IL-6 and CXCL9 (24). CXCL9 facilitates the recruitment of neighboring T cells to the microenvironment early after onset (25). IL-6 reinforces macrophage senescence in an autocrine manner and induces senescence in a subpopulation of fibroblasts (26). These fibroblasts, affected by macrophages, T cells, and senescence, could also synthesize excessive abnormal collagens and extracellular matrix components, resulting in LG fibrosis. Another SASP factor, osteopontin, can promote epithelial-mesenchymal transition (EMT), another fibroblast source, in the cGVHD-affected LG, participating in the destruction and fibrosis of the LG matrix (27). It has been reported that EMT can lead to reduced expression of vesicle-associated membrane protein 8 (VAMP8), which is one of the soluble N-ethylmaleimide-sensitive factor attachment protein receptor proteins and promoting the completion of autophagy by regulating the binding of autophagosomes and lysosomes during autophagy (28, 29). Fukui et al (28) hypothesized that decreased expression of VAMP8 affects the exocytosis of secretory vesicles in LG epithelia, leading to dry eye. Thus, a continuing investigation into the relationship between VAMP8 expression and EMT-related molecules is required to gain greater insights into the process of ocular GVHD. Additionally, the accumulation of lipofuscin-like inclusions in the LG of cGVHD patients as a result of increased oxidative stress may be influenced by the accelerated aging process. Lipofuscin accumulation causes oxidative damage to acinar cells, causing decreases in tear production (30). Collectively, in ocular GVHD, these alterations ultimately result in dryness and keratoconjunctivitis sicca **(** **Figure 2** **)**.

The efficacy of artificial tears for the first-line management of ocular GVHD patients with dry eyes has been confirmed. It has been revealed that artificial tears lubricate the ocular surface, replenish tears, and even decrease ocular surface inflammation (31). Nevertheless, due to cytotoxicity and the effects of insoluble crystalline calcium phosphate on the damaged corneal surface, it is recommended to cautiously use products containing preservatives and a high concentration of phosphate (32, 33). In patients with severe LG, dysfunction may derive additional benefits from reversible punctual occlusion with a silicone plug or permanent occlusion with thermal cauterization to reduce tear drainage (34, 35).

As shown above, fibrosis is another prominent pathological hallmark of ocular GVHD, and HSP47-expressing fibroblasts play a critical role in LG fibrosis. Reducing collagen synthesis by inhibiting the expression of HSP47 in fibroblasts may contribute to antifibrotic effects. In animal models of ocular GVHD, various studies related to this novel strategy against LG fibrosis are in progress. In a mouse model of ocular chronic GVHD, vitamin A–coupled liposomes containing HSP47 small interfering RNA against HSP47 (VA-lip HSP47) reduced HSP47 expression in fibroblasts, decreased collagen deposition, and restored tear secretion after ocular instillation (36). Another inhibition experiment revealed that the intraperitoneal administration of an AT1R antagonist (valsartan) to chronic GVHD mouse model suppressed fibrosis and prevented the development and progression of LG fibrosis. According to the results, the researchers proposed two related mechanisms. First, valsartan may ameliorate fibrosis by directly reducing HSP47 expression and collagen. Second, valsartan may suppress the number of fibroblasts expressing high levels of HSP47 and collagen, resulting in decreased HSP47 expression and collagen (22). In addition, Ogawa et al. (37) indicated that in mice, subconjunctival and subcutaneous injection of heavy chain-hyaluronan/pentraxin 3, purified from the human amniotic membrane, preserved tear secretion and conjunctival goblet cell density and mitigated the inflammation and fibrosis to reduce conjunctiva scarring. As shown above, indicators for the endoplasmic reticulum stress and activation markers for fibroblasts were elevated in cGVHD-affected LG fibroblasts. Hence, 4-phenyl butyric acid reduced cGVHD-induced endoplasmic reticulum stress and thereby alleviate inflammation and fibrosis to be a clinically translatable method to treat ocular GVHD (21). Previously, it was reported that tranilast has been used to treat scleroderma and other skin disorders by inhibiting the actions of transforming growth factor-β (TGF-β), a profibrotic growth factor that is pathogenically related to the excessive accumulation of collagenous matrix (38). Both topical tranilast in a clinical trial and oral tranilast in an animal trial showed evidence of being effective in alleviating inflammation and retarding fibrotic changes in LG pathology in cGVHD by suppressing the expression and/or activation of thioredoxin interaction protein and nuclear factor kappa-light-chain-enhancer of activated B cells and preventing EMT (39, 40). In the future, it will be necessary to conduct studies with larger sample sizes and randomized, controlled clinical trials. Owing to the potential association between ocular GVHD and senescent cell accumulation, ABT-263 may be a new therapeutic option by specifically eliminating senescent cells. Reportedly, ABT-263 specifically inhibited the antiapoptotic proteins (BCL-2 and BCL-xL) to reduce the detrimental effects of senescent cells on ocular GVHD (24).

### 2.2 Corneal impairment

**Figure 3:**
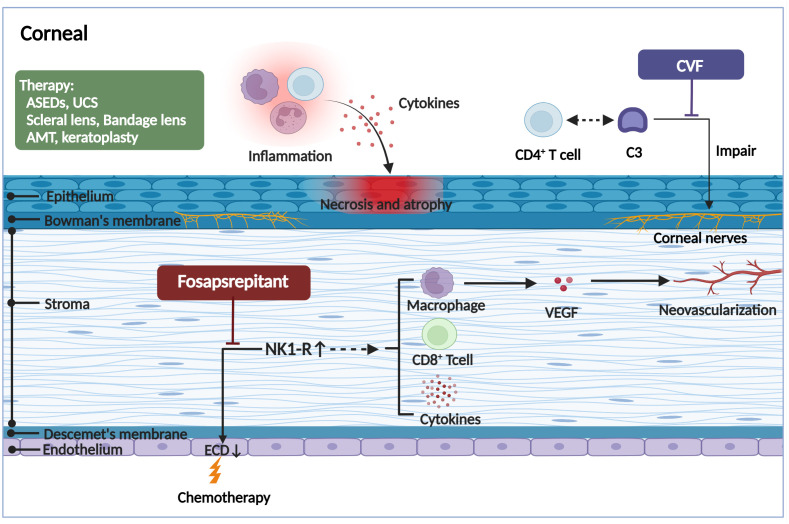
The pathogenic mechanism in the corneal of ocular GVHD and treatment. The corneal may appear the corneal epithelial impairment, a lower endothelial cell density, hemangiogenesis, and corneal neurosensory abnormalities in ocular GVHD. The corneal epithelial impairment including atrophy and necrosis, the vacuolization of epithelial basal cells, and stromal edema, all of which are likely associated with the infiltration of immune cells and the stimulation of inflammatory cytokines. The reduction of the corneal endothelial cells and formation of neovascularization might be associated with increased expression of the proinflammatory marker NK1R. Fosaprepitant, the inhibition of the SP-NKR1 axis, through topically applied substantially ameliorated the clinical manifestations of ocular GVHD. In addition, the aberrant complement C3/CD4+ T-cell axis activation might coordinate corneal nerve damage. However, the specific mechanism needs to be further investigated. By forming a biochemically stable convertase to rapidly hydrolyze mammalian C3, localized cobra venom factor prevents corneal sensation loss in ocular GVHD. In addition, the therapies for ocular GVHD patients with severe corneal impairment include serum eye drops, umbilical cord serum, scleral lenses, bandage soft contact lenses, amniotic membrane grafts, and keratoplasty. ASEDs, autologous serum eye drops; UCS, umbilical cord serum; AMT, amniotic membrane graft; CVF, cobra venom factor; ECD, endothelial cell density; NK1R, neurokinin-1 receptor; VEGF, vascular endothelial growth factor. (Created with BioRender.com).

Due to the severe inflammatory reaction and fibrosis in the LG, meibomian gland, and eyelid in patients with ocular GVHD, the cornea also often suffers secondary complications. Typically, patients present with pain, photophobia, vision impairment, and high-frequency application of tear substitutes, which significantly affects the patient’s activities of daily living (41). Corneal epithelial damage can be observed under slit lamp examination, manifesting as punctate keratopathy, filamentous keratitis, and persistent epithelial defects. In severe cases, corneal epithelial damage may also lead to corneal ulceration or even peripheral perforation with deficient healing and keratoconus formation, corneal neovascularization, limbal stem cell deficiency, and subsequent infection (42). In multiple ocular GVHD animal models, corneal alterations may also appear as corneal epithelial atrophy and necrosis, the vacuolization of epithelial basal cells, and stromal edema (43), all of which are likely associated with macrophages, donor-derived T-cell infiltration, inflammatory cytokines, and protein hydrolases such as matrix metalloproteinases (MMPs) (44). In addition, inflammation associated with ocular GVHD may involve all deeper corneal layers up to the endothelium, not just the surface epithelium. Before HSCT, patients exhibited a lower endothelial cell density (ECD) than healthy controls. After HSCT, the ECD further significantly decreased, particularly in patients who developed ocular GVHD (45). A loss of corneal endothelial cells but an upregulation of neurokinin-1 receptor (NK1R) was observed in an acute GVHD murine model, suggesting that ocular GVHD affects the corneal endothelium, inducing the cell number reduction, which is associated with increased expression of the proinflammatory marker NK1R (46). NK1R is the principal receptor of pro-inflammatory neuropeptide Substance P (SP). Hence, it has been reported that the SP/NK1R pathway may be associated with a reduction in corneal endothelial cell quantity (47). Moreover, activation of NK1R can directly damage the endothelium by inducing CD8+ T lymphocyte cytotoxic activity and indirectly by inducing the secretion of tumor necrosis factor (TNF)-α, interferon (IFN)-γ, interleukin (IL)-1α, and IL-1β (48, 49). NK1R activation also promotes vascular endothelial growth factor (VEGF) secretion from macrophages and stimulates hemangiogenesis and lymphangiogenesis, thereby amplifying the inflammatory response (50). Nonetheless, additional research is required to elucidate the precise mechanisms that lead to corneal endothelial loss. In addition, corneal neurosensory abnormalities can be seen early in patients with ocular GVHD. It has been found that aberrant complement C3/CD4+ T-cell axis activation can coordinate corneal nerve damage, leading to neurotrophic ulcers (51) **(** **Figure 3** **).**

In addition to the use of autologous serum eye drops (ASEDs) as tear replacements, wound healing factors such as TGF-β, nerve growth factor, epidermal growth factor (EGF), and fibroblast growth factor, which are present in ASEDs in abundance, support the epithelial healing of severely impaired corneas and conjunctivae (52, 53). Several researchers have confirmed that ASEDs was effective in treating severe cases of ocular GVHD at dilutions ranging from 20% to 100% (54, 55). However, several open questions remain, including the possible risk of superimposed infection, the side effects of long-term application, the effects of systemically applied immune suppressive drugs in serum, and so on (56). In addition, autologous serum may be unavailable in patients with poor venous access or coexisting systemic diseases, such as anemia and blood dyscrasia (57). As alternatives, allogeneic serum, umbilical cord serum, and platelet-derived eye drops offer alternative therapeutic options to patients who cannot provide autologous serum (58–60). However, caution must be exercised when administering allogeneic serum to avoid the risk of bloodborne diseases and allergic reactions (61). For platelet-derived eye drops, two clinical trials related to platelet lysate are in progress (NCT05311514, NCT03414645). Overall, blood-derived products are promising future therapies to treat patients with ocular GVHD.

Additionally, scleral lenses and bandage soft contact lenses can be used to treat ocular GVHD with a severely refractory ocular surface (62, 63). When severe keratitis sicca is resistant to treatment or is complicated by persistent epithelial defects, an amniotic membrane graft can be used to promote epithelialization, suppress inflammation, and reduce subsequent scarring (64–66). Simultaneously, limbal epithelial transplantation and keratoplasty have also been reported in patients with ocular GVHD (67). However, the prognosis for these patients is grim because corneal transplantation is considered high-risk in ocular GVHD due to the presence of severe inflammation.

Due to the finding that NK1R is relevant to ocular GVHD, Romina et al. (47) discovered that inhibition SP-NKR1 axis with topically administered fosaprepitant substantially ameliorated the clinical course of ocular GVHD. Fosaprepitant reduced not only the corneal fluorescein staining score (by 72%) but also inflammation in the conjunctiva and LG, which indicates that NK1R is a novel and promising druggable target to regulate immune dysregulation in ocular GVHD. In addition, fosaprepitant appears to be ideally suited for topical administration due to its safety and high water solubility (50). Hence, prompt administration of fosaprepitant at the onset of initial clinical manifestations could prevent immune cell recruitment and reactivation, ultimately reducing tissue damage caused by alloreactive T cells. Identifying the role of the complement pathway in initiating corneal sensation involvement in ocular GVHD is a significant step forward in the mechanistic understanding of this disease process, as it may represent a novel aspect and treatment target. It is reported that localized cobra venom factor (CVF) treatment prevents corneal sensation loss in ocular GVHD (51). Purified CVF, a ‘nontoxic’ derivative, forms a biochemically stable convertase to rapidly hydrolyze mammalian C3, which is the heart of the pathway. While topical CVF administration did not induce respiratory or corneal abnormalities in any animals, differences in complement regulation exist between mouse models and humans that must be resolved in order to further evaluate the therapeutic potential of CVF in ocular GVHD patients.

### 2.3 Conjunctival involvement

**Figure 4:**
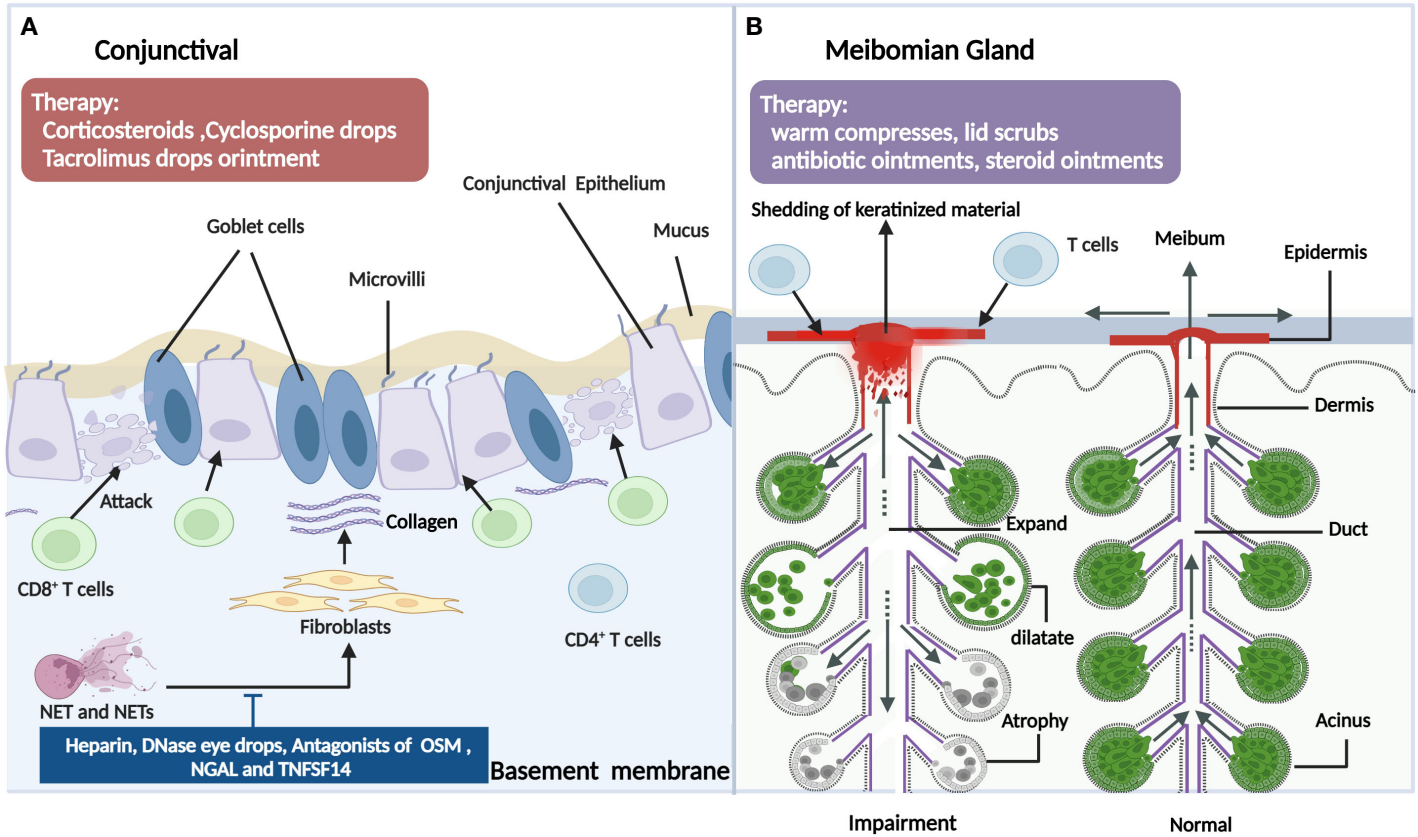
The pathogenic mechanism in the conjunctival **(A)** and meibomian gland **(B)** of ocular GVHD and treatment. 4A: In ocular GVHD patients, the conjunctival often presents as the conjunctival injection or chronic conjunctivitis, which is related with various immune cells including T cells and neutrophils (NET). By releasing nuclear chromatin complexes as extracellular DNA webs which are termed neutrophil extracellular traps (NETs), Net and Nets cause conjunctival fibroblast proliferation and differentiation and may contribute to conjunctival fibrosis. Hence, drugs such as deoxyribonuclease I; a subanticoagulant dose of heparin; and antagonists of OSM, NGAL and TNFSF14 could be potential therapies for managing ocular GVHD. In addition, more metaplasia, fewer goblet cells, a lower mean number of mucosal microvilli, and shorter microvilli also contribute to dry eye in ocular GVHD. For ocular GVHD patients with conjunctival Involvement, the therapies also include corticosteroids, cyclosporine drops, tacrolimus drops, or ointment. 4B: Infiltration of the lymphocytes results in damage to the meibomian gland in ocular GVHD. In addition, the meibomian gland in ocular GVHD appears cystic dilatation with atrophy because of ductal epithelial hyperkeratinization, the shedding of keratinized material into the glandular ducts leading to obstructions of the orifice. Patients with ocular GVHD also develop absent meibomian glands, resulting in tear film instability. However, there is a lack of suitable animal models to explore the related mechanism of meibomian gland involvement in ocular GVHD to find specific therapeutic targets. The clinical usual treatment includes warm compresses, lid scrubs, antibiotic ointments, and steroid ointments for ocular GVHD patients with meibomian gland dysfunction. OSM, oncostatin M; NGAL, neutrophil gelatinase-associated lipocalin; TNFSF14: tumor necrosis factor superfamily member 14 (Created with BioRender.com).

The conjunctival involvement of patients with ocular GVHD often presents as a conjunctival injection or chronic conjunctivitis. The typical histopathological presentation is accompanied by lymphocytic infiltration of the subconjunctival stroma (68). Tatematus et al. (69) reported a large infiltration of CD8+ T cells in the basal surface of the conjunctiva in patients with cGVHD-associated dry eye, which damaged the basal cells to cause abnormal regeneration of the conjunctival epithelia and impair exocrine function. In an animal model of GVHD, in addition to the LG, interactions between T cells in the conjunctiva and mesenchymal stem cells also contributed to the progression of dry eye disease (DED) (70). Another major histological finding of the conjunctiva in ocular GVHD has marked fibrosis of the subepithelial mesenchyme (71). Conjunctival subepithelial fibrosis also referred to as subtarsal fibrosis, is present in 50% of patients with ocular GVHD but is absent in all non-GVHD moderate or severe DED patients. Animal studies have also shown that GVHD-associated conjunctival fibrosis is accompanied by donor-derived myofibroblast formation and Ras system activation (72–74). Additionally, there was marked infiltration of neutrophils at the upper palpebral conjunctiva in patients with ocular GVHD, which might be associated with the clinical manifestations and inflammatory status of the ocular surface. Neutrophils release nuclear chromatin complexes as extracellular DNA (eDNA) webs that are termed neutrophil extracellular traps (NETs) (75). It has been reported that NETs can cause conjunctival fibroblast proliferation and differentiation, which may contribute to conjunctival fibrosis. Moreover, NETs and NET-associated proteins also contribute to other pathological changes in ocular GVHD, including corneal epitheliopathy, ocular surface inflammation, and meibomian gland disease (76). Furthermore, the conjunctival mucosal microvilli of ocular GVHD patients differed significantly in number and morphology from healthy individuals and Sjogren’s syndrome patients. The ocular GVHD conjunctivae demonstrated significantly more metaplasia and fewer goblet cells, a lower mean number of mucosal microvilli, and shorter microvilli. The average number of secretory vesicles is also significantly lower, and the membrane-spanning mucin is thinner (69, 77). Consequently, these may be significant factors influencing the stability of the tear film layer and its contribution to cGVHD-related dry eye. In addition, due to the decrease of goblet cells and the mucin layer and other factors, ocular surface microbes were showed more diverse in the ocular GVHD patients compared with non-ocular GVHD patients (78). However, A prospective study reported a different result in which the microbiome on the ocular surface was characterized by a loss of diversity in ocular GVHD patients (79). Both investigations indicated that the ocular surface microbial dysbiosis is involved in the underlying microbial mechanism of ocular GVHD, but further investigations are warranted to explore the concrete mechanisms and therapeutic targets (80). Other less prevalent characteristics include cicatricial conjunctivitis (a kind of chronic conjunctivitis with conjunctival fibrosis and scarring formation), cicatricial entropion(inversion of the eyelids caused by fibrosis and scarring of the eyelid), symblepharon(any adhesion between the palpebral and bulbar conjunctiva), ankyloblepharon (partial or complete adhesion of upper and lower eyelids), and lagophthalmos(an inability to close the eyelids), which could progress to conjunctival keratinization and punctual occlusion (26). Recently, superior limbal keratoconjunctivitis(SLK)-like inflammation was reported as a manifestation in a cohort of ocular GVHD patients, characterized by the inflammation and staining of the superior conjunctiva and the alteration of the superior limbal epithelium with corneal filaments (81, 82). SLK may aggravate limbal stem cell deficiency and corneal pannus formation and may be correlated with the degree of upper eyelid laxity (83) (**Figure 4A** **)**.

Ocular surface chronic inflammation is essential to ocular GVHD pathogenesis, and topical anti-inflammatory drugs, including corticosteroids, cyclosporine A (CSA), and tacrolimus, have already been employed to treat patients with ocular GVHD.

Topical steroids have the capacity to promote lymphocyte apoptosis and suppress cell-mediated inflammation (84). A complete response was documented in all seven patients with progressive cicatricial conjunctivitis associated with ocular GVHD treated with prednisolone acetate 1% eye drop therapy for a total treatment duration of 7 weeks (85). In addition, pre-HSCT initiation with a loteprednol etabonate 0.5% ophthalmic suspension (LE 0.5%) may be effective for the treatment and prophylaxis of ocular GVHD and appears to be safe (86). In contrast, it has been reported that the low-dose topical steroid regimen LE 0.5% might decrease the favorable response in ocular GVHD patients (87). During drug administration, patients should also be monitored regularly for adverse effects, including increased intraocular pressure, cataracts, glaucoma, and infectious keratitis (88). Moreover, several new topical steroid regimens have been indicated to be safe and effective for ocular GVHD. Forehead application of 1% progesterone gel also improved ocular signs and symptoms without severe adverse events, potentially revealing a novel neuroaxis drug delivery mechanism (89). Two relevant clinical studies are being activated (NCT03990051) and recruiting patients (NCT04769648).

CSA is a calcineurin inhibitor that exerts immunomodulatory effects by blocking T-cell infiltration and activation and the subsequent release of inflammatory cytokines and increases goblet cell density to improve ocular surface and tear functions (90). Topical CSA may also be an effective prophylactic and therapeutic measure for dry eye patients with cGVHD (91, 92). According to the study, dry eye symptoms improved in 62.5% of patients with ocular GVHD treated with topical CSA, and the corneal fluorescein staining score improved in all eyes (P = 0.0039) (93). Due to the underlying cause of severe keratitis, there are differences in tolerance between different CSA formulations. Among these, cationic 0.1% CSA is considerably less tolerated. Common intolerable symptoms include burning sensations, redness, conjunctival and lid swelling, and itching (94). Tacrolimus, with a mechanism of action similar to that of cyclosporine, is more effective than CSA (95). Research has shown the efficacy and safety of topical tacrolimus in controlling ocular surface inflammation when conventional topical treatments have failed or the patient cannot tolerate topical CSA (96, 97). Otherwise, Abud et al. (98) observed no significant difference between the composite tolerability scores of topical tacrolimus and methylprednisolone in ocular GVHD patients (P=0.06), while topical tacrolimus was more effective than methylprednisolone in reducing the corneal fluorescein staining score at week 10 (55% and 23% reduction, respectively; P=0.01). However, the burning sensation was more pronounced with tacrolimus (P=0.002). Furthermore, a registered clinical trial for the evaluation of 0.05% CSA and 0.1% tacrolimus has been completed, but the results are not published (NCT05294666).

As shown above, NETs and NET-associated proteins, including eDNA, oncostatin M (OSM), neutrophil gelatinase-associated lipocalin (NGAL), and tumor necrosis factor superfamily member 14(TNFSF14) have the potential to cause ocular surface pathology in ocular GVHD patients. Hence, drugs such as deoxyribonuclease I (DNase I); a subanticoagulant dose of heparin; and antagonists of OSM, NGAL, and TNFSF14 could be potential therapies for managing ocular GVHD (76). Among these, a NET-dismantling biologic, a subanticoagulant dose of heparin (100 IU/mL), serves as a treatment for ocular GVHD. In addition to destroying NETs, low-dose heparin has independent immunosuppressive and anti-inflammatory effects and is antifibrotic and corneal epithelium nontoxic. However, the specific mechanism of low-dose heparin on ocular GVHD requires more investigation. Notably, heparin might cause corneal stromal and subconjunctival tissue hemorrhage (99). In addition, DNase eye drops (75, 100) and human intravenous immune globulin (IVIG) eye drops (4 mg/mL) (101) both appear safe and well tolerated and have the potential to lessen the severity of signs and symptoms of DED in patients based on previous studies. The clinical study about IVIG eye drop treatment for DED is ongoing (NCT03992482).

In addition, the treatment for systemic GVHD based on the microbiome, in the animal model by oral various antibiotics, revealed that gentamicin significantly suppressed inflammatory cell infiltration and fibrosis in cGVHD-affected organs and attenuate the ocular manifestations of cGVHD (102).

### 2.4 Meibomian gland dysfunction

Meibomian gland dysfunction (MGD) is the second most frequent complication of ocular GVHD, with a prevalence of 47.8% in this disease (103). Although the pathogenesis of MGD is still vague, some morphological and functional alterations in the meibomian glands are shown in ocular GVHD. In a histological study, ductal epithelial hyperkeratinization, the shedding of keratinized material into the glandular ducts leading to obstructions of the orifice, and ultimately cystic dilatation with atrophy were found in MGD (104). Moreover, ocular GVHD was characterized by ductal epithelial destruction due to lymphocyte aggregation, the sloughing of epithelial cells due to lymphocyte infiltration or pseudomembrane formation, and eventual extensive fibrosis around the orifice, ductules, ducts, and acini of the meibomian gland (103, 105–107). These results suggest that ocular GVHD patients may exhibit a combination of aqueous deficiency and evaporative DED, consequently demonstrating more severe ocular discomfort and worse ocular surface conditions. In addition, MGD in ocular GVHD patients is probably associated with anatomical changes. Loss of the meibomian gland diminishes the quantity and quality of meibum, consequently leading to abnormal lipid secretion by meibomian glands, tear film instability and decreased tear breakup time, and even cornea and conjunctiva changes (108–110). The mean meibomian gland acinar unit density value was lower and the mean acinar diameter was shorter in the ocular GVHD group compared to that without ocular GVHD (103). A single-center retrospective study showed that a linear relationship between the percentage of the meibomian gland acinar area and the severity of ocular GVHD. It was indicated that the more severe the ocular cGVHD was, the smaller the percentage of the meibomian gland acinar area, and the more obvious the meibomian gland loss (111). Moreover, eyelid margin abnormalities were accompanied by meibomian gland damage in the course of ocular GVHD. In addition to the atrophy and irregularity of the eyelid margins, lash loss, lacrimal puncti stenosis, and cicatricial upper eyelid entropion may aggravate ocular surface damage and worsen ocular discomfort in ocular GVHD (112–114). Due to the lack of suitable preclinical animal models and sensitive strategies or approaches to studying the condition, the involvement of the meibomian glands and their dysfunction in the development of ocular GVHD is poorly understood. Therefore, there is a need for deeper insights into the pathogenic mechanism of ocular GVHD, in particular the effect of HSCT on the meibomian glands and their role in ocular GVHD, through the use of appropriate preclinical models and sound research strategies **(** **Figure 4B** **)**.

In patients with meibomian gland dysfunction, which causes tear film instability and evaporative dry eye, the usual treatment lines include warm compresses, lid scrubs, and the maintenance of proper lid hygiene practices (115). In particular, daily or twice daily application of warm compresses to the eyelids followed by gentle massage is a low-cost and simple-to-implement intervention for enhancing tear film quality and reducing tear evaporation (116). Additionally, antibiotic ointment or eye drops can be applied for bacterial lid margin superinfection (84). It has been reported that ophthalmic steroid ointments can be applied to the lid margins for ocular GVHD-associated blepharitis treatment, with an overall positive response rate of 97.2% (117). Furthermore, nutritional supplements such as fish oil (omega−3 fatty acids) and flaxseed oil (2000 mg/d) may be beneficial due to their anti−inflammatory properties (118). In patients with severe dry eyes, partial Tarsorrhaphy, a procedure for closure of the lid fissure and protection of the cornea, may be important to decrease the exposed area of the corneal surface (11).

### 2.5 Other new therapies

Several other promising novel treatments for ocular GVHD have demonstrated efficacy in reducing inflammation and halting disease progression. In a preclinical mouse model, a vascular adhesion protein-1 inhibitor (119), a secondary lymphoid-tissue chemokine (CCR ligand 21) antagonist (120), and subconjunctival injection of AAV-HLA-G (a therapy of adeno-associated virus (AAV) gene transfer of HLA-G) (121) served as effective and safe therapeutic agents to reduce inflammation and ameliorate clinical signs of ocular GVHD. However, these need to be registered for clinical use. Recently, inhibiting Janus kinase (JAK) family members (including JAK1, JAK2, JAK3, and tyrosine kinase 2) and spleen tyrosine kinase (SYK) in immune cells are gaining interest as a means to suppress inflammation. Previously, it was demonstrated that entospletinib, a second-generation highly selective SYK inhibitor, could improve blood immune cell reconstitution, prolong survival and improve clinical eye scores in GVHD mice (122). Ruxolitinib, a JAK 1/2 inhibitor, has been used to treat steroid-resistant or steroid-dependent cGVHD patients, and ocular GVHD significantly improved after ruxolitinib treatment (123, 124). A randomized pilot trial also indicated that the 0.5% concentration of R348, a topical combined JAK/SYK inhibitor, was an effective corneal epitheliopathy treatment in patients with ocular GVHD (125).

At present, the use of cell-based therapy is a potential therapeutic strategy for the treatment of various conditions (126). Given the unique immunomodulatory properties of mesenchymal stromal cells (MSCs), it was discovered that MSCs infusion might inhibit cGVHD symptoms (127), and 54.55% of patients showed an improvement in both ocular symptoms and Schirmer’s test results (128). In an experimental model of ocular GVHD, it was also confirmed that treatment with a subconjunctival injection of human MSCs (hMSCs) is effective in reducing corneal inflammation and squamous metaplasia in ocular GVHD (129). Recently, in a prospective clinical trial, 28 eyes with refractory GVHD–DED exhibited substantial relief after treatment with exosomes from MSCs, showing reduced fluorescein scores, longer tear breakup time, increased tear secretion, and lower OSDI scores (130). Regulatory T cells (Tregs) have a critical role in the immune system by maintaining immune homeostasis and preventing the occurrence of autoimmune diseases (131, 132). It has been demonstrated that early post-transplantation treatment of patients with expanded Tregs + BETi (a bromodomain and extraterminal protein inhibitor) improved the level of Tregs and exhibit a significant diminution of GVHD clinical scores with less ocular involvement (133). However, experiments using animal models of GVHD are needed to evaluate the potential role of Tregs as an innovative approach to overcoming ocular GVHD.

In the clinical trial phase, long-term treatment with topical rebamipide and diquafosol can improve clinical signs and symptoms by enhancing tear stability and ocular surface condition due to their mucin-inducing and secretion-promoting effects (134, 135). According to reports, the interaction between lymphocyte function-associated antigen-1 (LFA-1) and intercellular adhesion molecule (ICAM-1) was involved in the trafficking of alloreactive lymphocytes to GVHD target organs, the activation of T cells, and subsequent immune (alloreactive) cell-mediated tissue damage (136). It was observed that the use of lifitegrast, an LFA-1 direct competitive antagonist that works by blocking the interaction between ICAM-1 and LFA-1, significantly improved NIH severity scores in 44% of patients (137). However, ophthalmologists and hematologists should be aware of the potentially severe side effects of lifitegrast, which could result in corneal infection and perforation (138). In addition, there are several ongoing or recently concluded clinical trials evaluating novel therapeutics for ocular GVHD, including amniotic fluid eye drops (NCT03298815), Vigamox (NCT04204122), brimonidine tartrate (NCT03591874) and umbilical MSCs derived exosomes (NCT04213248) **(** **Table 1** **)**.

**Table 1:** Registered clinical trials of ocular GVHD (from January 2018 to September 2022).

Interventions	Agent	Pts	Phase	Status	Institutions	Locations	Trial number
**Drug**	Cyclosporine vs. tacrolimus	89	Phase 4	Completed	Peking University Third Hospital	Beijing, China	NCT05294666
	Cyclosporine ophthalmic (Ikervis)	40	Phase 4	Recruiting	Singapore Eye Research Institute	Singapore, Singapore	NCT04636918
	Brimonidine tartrate	59	Phase 3	Terminated	Mayo Clinic Phoenix, Arizona	United States	NCT03591874
	Vigamox	30	Phase 2	Recruiting	Washington University School of Medicine Saint Louis	Missouri, United States	NCT04204122
	Pro-ocular™ topical gel 1%	38	Phase 2	Recruiting	Boston Sight Needham	Massachusetts, United States	NCT04769648
	Intravenous immune globulin (IVIG)	27	Phase 1	Completed	Illinois Eye and Ear Infirmary	Chicago, Illinois, United States	NCT03992482
	Lifitegrast 5% ophthalmic solution	30	Early Phase 1	Recruiting	Richard W Yee, MD PLLC Bellaire	Texas, United States	NCT04792580
**Biological**	UMSC-derived exosomes	27	Phase 1	Recruiting	Zhongshan Ophthalmic Center Guangzhou	Guangdong, China	NCT04213248
	Allogeneic platelet lysate eye drops	30	Phase 2	Recruiting	First Pavlov State Medical University of Saint Petersburg	Saint Petersburg, Russian Federation	NCT05311514
	Amniotic fluid eye drops (AFEDs)	15	Phase 1	Recruiting	University of Utah Huntsman Cancer Institute	Salt Lake City, Utah, United States	NCT03298815
	CAM-101 10%	64	Phase 1	Completed	Byers Eye Institute of Stanford University	Palo Alto, California, United States	NCT03414645
**Device**	Tangible Boost	50	Not Applicable	Recruiting	Boston Sight Needham	Massachusetts, United States	NCT04313725

UMSC, umbilical mesenchymal stem cell, CAM-101 10, topical fibrinogen-depleted human platelet lysate in patients.

## 3 Ocular GVHD biomarkers

Cellular mediators also play a crucial role in the underlying mechanism of pathophysiological processes. Consequently, it is essential to investigate the function and cellular origin of each soluble mediator in the tear film that helps to improve the ocular surface condition in GVHD-associated DED. In addition, tear testing is a simple, safe, quantifiable noninvasive screening method. Previously, various studies have identified tear cytokines/chemokines, lipid metabolites, and proteins as promising potential objective biomarkers that could serve as diagnostic, prognostic, and monitoring tools for both ocular and systemic diseases **(** **Table 2** **)**.

**Table 2:** Summary of data from the studies investigating tear molecules in ocular GVHD.

Analysis methods	Control group	Pts	Tear collection	Results	Ref
Immunoassay	DED	23	Tear collector	IL-1β ↑, IL-6↑, IL-8 ↑, ICAM-1↑, IL-7 ↓, EGF ↓	(139)
	Non ocular GVHD	20	Micropipette	IL-8↑, MIP-1α↑	(140)
	Non ocular GVHD and Healthy control	34	Schirmer strips	IL-6 ↑, IFN-γ↑	(141)
	Non ocular GVHD and Healthy control	32	Schirmer strips, Capillary tubes	IFN-γ ↑, IL-6 ↑, IL-8 ↑, IL-10 ↑, IL-12AP70 ↑, IL-17A ↑, MMP-9 ↑, VEGF ↑	(142)
	Healthy control	22	Capillary tubes	IL-1Ra ↑, IL-8/CXCL8 ↑, IL-10 ↑, EGF↓, IP-10/CXCL10 ↓	(143)
	Healthy control	22	Capillary tear collector	LT-α ↓	(144)
	DED	18	Capillary tear collector	IL-2↑, IL-6↑, IL-8↑, ICAM-1 ↑, CD62E ↑, Neuropilin-1↑, MMP-3↑, BAFF ↑	(145)
Proteomics	DED	45	Single-use test card	MMP-9↑	(146)
	Healthy control	14	Micropipette	NE ↑, MMP-8↑, MMP-9↑, MPO↑	(147)
	Non ocular GVHD and Healthy control	48	Microcapillary tubes	NE ↑, MPO ↑, IL-8↑, oncostatin M ↑, TNFSF14 ↑, TNF-α↑, BDNF ↑	(76)
	Non ocular GVHD	10	Schirmer strips	Nucleic acid binding↑, Cytoskeletal proteins ↑, Transfer and receptor proteins ↓, Enzyme modulators ↓, Hydrolases↓	(148)
Lipid metabolites	Healthy control	23	Capillary tear collector	Phosphatidylcholines: PC (34:1), PC (34:2), PC (36:2), and PC (10:0/22:0) ↑	(149)
				Sphingolipid: serine, sphingomyelin, and glucosylceramide ↑	
				Unsaturated fatty acids: docosahexaenoic acid (DHA) and palmitic acid ↑	

IL, interleukin; ICAM-1, intercellular adhesion molecule-1; EGF, epidermal growth factor; IFN-γ, interferon gamma; MMP-9, Matrix Metalloproteinase-9; VEGF, vascular endothelial growth factor; LT-α, lymphotoxin-α; CD62E, E-selectin; BAFF, B-cell activation factor; NE, Neutrophil Elastase; MPO, myeloperoxidase; TNFSF14, tumor necrosis factor superfamily member 14; BDNF, Brain-derived neurotrophic factor. The symbols ↑ means “increased” and the symbol ↓ means “decreased”.

In ocular GVHD, T cells have long been recognized as a key driver of alloreactivity. Hence, various cytokines related to T helper cell-1, T helper cell-2, or T helper cell-17 are involved in this immune process. Previous studies have characterized the tear cytokine profile of patients with ocular GVHD. In particular, it was found that IL-1, IL-2, IL-6, IL-8, IL-10, IL-17, IP-10/CXCL-10, ICAM-1, TNF-α, EGF, lymphotoxin-α(LT-α), E-selectin (CD62E), B-cell activation factor (BAFF), and neuropilin-1 could be regarded as biomarkers of ocular GVHD for prediction, diagnosis, and prognosis (139, 142, 145, 150–152). In addition to their clinical utility, these biomarkers can identify potential therapeutic mechanisms and biological targets (153). For prediction, Cocho et al. (143) generated predictive models based on the best panel of IL-8/CXCL8 and IP-10/CXCL10 tear levels along with age and sex, which showed good sensitivity (86.36%) and specificity (95.24%). Ma et al. (144) found that tear LT-α levels below 0.203 ng/mL could be used to predict the presence of ocular GVHD. For diagnosis, Shen et al. (145) showed that a proliferation-inducing ligand (APRIL)/BAFF had superior diagnostic capabilities, revealing that B cells may play a crucial role as immune substrates in the immune process of ocular GVHD and providing direction for further B-cell mechanism research (154). In addition, some tear cytokines, such as IL-6, IL-8, IL-10, IFN-γ, TNF-α, and EGF, were correlated with clinical ocular surface parameters including OSDI, Schirmer’s score, the corneal fluorescein staining score and tear breakup time, and contributed to the assessment of the severity of ocular GVHD (139–141, 143, 151). It was found that a model based on pre-HSCT tear levels of the inflammatory molecules fractalkine, IL-1Ra, and IL-6 had a good prognostic ability for the development of ocular cGVHD (152). Hence, pre-HSCT tear cytokines could also potentially serve as susceptibility biomarkers for the development of ocular GVHD after HSCT. Furthermore, tear cytokines are also associated with therapeutic effects in ocular GVHD. Ma et al. (155) revealed that tear IL-6 and IL-8 levels were significantly altered in response to effective ocular GVHD therapy, which helped to provide a more integrated picture of the response and resistance mechanisms in ocular GVHD and a deeper understanding of these mechanisms to enhance the ocular GVHD treatment efficacy.

Profiling the proteomics and lipid metabolites of tear fluid from GVHD patients may also reveal a potential biomarker signature for the disease. Previous research has reported reduced levels of tear proteins in ocular GVHD. Gerber-Hollbach et al. (148) made additional efforts to investigate the proteomic profile of tears in ocular GVHD and identified 79 proteins whose expression was significantly different from that of non-ocular GVHD. Among them, histone H2B, Ig gamma 1 chain C region, periplakin, prelamin-A/C, and ribosome binding protein 1 were the most prominently upregulated, whereas lactotransferrin, extracellular glycoprotein lacritin, proline rich protein, lipocalin-1, and cystatin-S were the most significantly downregulated proteins. Nevertheless, additional research on correlations with clinical parameters is required to identify potential tear biomarker candidates for ocular GVHD. O’Leary et al. (156) compiled a list of 12 proteins significantly differentially expressed with advancing disease severity and discovered that the expression levels of a 13-marker tear protein panel in mild ocular GVHD may predict the development of more severe ocular GVHD clinical phenotypes. Lysozyme C, polymeric immunoglobulin receptor, and phosphoglycerate mutase 1 were significantly correlated with ocular surface parameters of ocular GVHD in this study. Additionally, neutrophils, exfoliated epithelial cells, NETs, and NET-associated proteins, such as MMPs, myeloperoxidase (MPO), neutrophil elastase (NE), eDNA, brain-derived neurotrophic factor (BDNF), OSM, NGAL, and TNFSF14, are elevated in ocular surface washings or mucocellular aggregates for ocular GVHD (146, 147). Specifically, NET-associated proteins (eDNA, OSM, NGAL, and TNFSF14) may be considered potential biomarkers for ocular GVHD (76). MMP-9 may be a reliable biomarker for differentiating transplanted patients from other forms of DED and facilitating the decision to initiate anti-inflammatory treatments and monitor their efficacy (146).

Ma et al. (149) analyzed the dysregulated patterns of the three lipid metabolic pathways and found a significant elevation of several phosphatidylcholines (PCs), serine, sphingomyelin (SM), lactosylceramide (LacCer), docosahexaenoic acid (Doco), and palmitic acid. Particularly, PC (34:1), SM, and LacCer were correlated with clinical parameters and could serve as potential biomarkers for the diagnosis and evaluation of ocular GVHD, as well as promising targets for its clinical treatment. This finding also suggested that the heightened immune response during ocular GVHD may be associated with lipid dysregulation, which necessitates further investigation into metabolic pathways.

## 4 Conclusion

Ocular GVHD, a common condition among patients after undergoing allo-HSCT, has a substantial negative impact on the quality of life of patients. Due to the complex pathological mechanisms and diverse clinical manifestations of ocular GVHD, it remains an obstacle to diagnosing affected patients. Moreover, there are still not enough satisfactory effective prophylactic and therapeutic strategies for ocular GVHD. Newer diagnostic methods, especially ocular surface biomarkers, could help in diagnosing the disease earlier, monitoring its response to treatment, and helping to further understand the occurrence and development of ocular GVHD. In addition, while some therapeutic strategies have demonstrated efficacy, the treatment is usually initiated during the symptomatic stage, and by that time, the damage to the LG and conjunctival tissue might be permanent. Therefore, it is particularly important to advocate prevention first, early diagnosis, and early intervention. It is also desirable to collect data on the long-term development and treatment outcomes of ocular GVHD by maintaining a regular follow−up of these patients to better support the implementation of preemptive and therapeutic strategies. Eventually, the elucidation and further study of the relevant pathogenic mechanism underlying ocular GVHD may translate into efficacious mechanism-based therapeutics to permit the development of new perspectives and targeted treatments for patients with ocular GVHD.

## Author contributions

The manuscript was conceptualized by XZ, XW, and RY. XC wrote the majority of the manuscript. XC, RH, and SH contributed equally to the manuscript. The figures were designed by RH and WF and drawn by XC and SH. XC, RH, and SH summarized the tables. All authors contributed to the article and approved the submitted version.

## Glossary

**Table:**

allo-HSCT	allogeneic hemaleftoietic stem cell transplantation
APCs	antigen-presenting cells
APRIL	a proliferation-inducing ligand
ASEDs	autologous serum eye drops
α-SMA	α-smooth muscle actin
BAFF	B-cell activation factor
BDNF	brain-derived neurotrophic factor
CD62E	E-selectin
cGVHD	chronic graft-versus-host disease
CVF	cobra venom factor
CSA	cyclosporine A
DED	dry eye disease
DNase I	deoxyribonuclease I
ECD	endothelial cell density
eDNA	extracellular DNA
EGF	epidermal growth factor
EMT	epithelial-mesenchymal transition
GVHD	graft-versus-host-disease
HSP	heat shock protein
ICAM-1	intercellular adhesion molecule-1
LE 0.5%	loteprednol etabonate 0.5% ophthalmic suspension
LFA-1	lymphocyte function-associated antigen-1
IFN	interferon
JAK	janus kinase
LacCer	lactosylceramide
LG	lacrimal gland
IL	interleukin
LT-α	lymphotoxin-α
IVIG	intravenous immune globulin
MGD	meibomian gland dysfunction
MMPs	metalloproteinases
MPO	myeloperoxidase
MSCs	mesenchymal stromal cells
NE	neutrophil elastase
NETs	neutrophil extracellular traps
NGAL	neutrophil gelatinase-associated lipocalin
NIH	National Institutes of Health
NK1R	neurokinin-1 receptor
OSDI	ocular surface disease index
OSM	oncostatin M
PCs	phosphatidylcholines
RAS	the tissue renin-angiotensin system
SASP	senescence-associated secretory phenotype
SLK	superior limbal keratoconjunctivitis
SM	sphingomyelin
SYK	spleen tyrosine kinase
TGF-β	transforming growth factor-β
TNF	tumor necrosis factor
TNFSF14	tumor necrosis factor superfamily member 14
Tregs	regulatory T cells
VAMP8	vesicle-associated membrane protein 8
VEGF	vascular endothelial growth factor
